# Perception Study of Traditional Korean Medical Students on the Medical Education Using the Dundee Ready Educational Environment Measure

**DOI:** 10.1155/2016/6042967

**Published:** 2016-11-28

**Authors:** Hyunho Kim, Hanyoung Jeong, Pyeongjin Jeon, Seungju Kim, Young-Bae Park, Yeonseok Kang

**Affiliations:** ^1^Department of Biofunctional Medicine and Diagnostics, College of Korean Medicine, Kyung Hee University, Seoul 02447, Republic of Korea; ^2^College of Korean Medicine, Wonkwang University, Iksan 54538, Republic of Korea; ^3^Department of Medical History, College of Korean Medicine, Wonkwang University, Iksan 54538, Republic of Korea; ^4^Institute of Korean Medicine Education and Evaluation, Seoul 07525, Republic of Korea

## Abstract

*Background*. In Korea, a few studies regarding traditional Korean medicine (TKM) education have been conducted. The aim of this study is to evaluate students' perceptions regarding TKM education in Korea and compare them with those of other countries using a quantitative scale, Dundee Ready Educational Environment Measure (DREEM).* Materials and Methods*. We conducted a survey using DREEM in a TKM college. Totally, 325 students responded to this survey and we performed the descriptive statistics of scores in all items, subscales, and total. Additionally, subgroup comparisons according to gender, school year, and academic achievement were analyzed.* Results*. Mean overall DREEM score was 94.65 out of 200, which is relatively low compared to previous studies. Particularly, perceptions regarding subscales of learning, atmosphere, and self-perceptions were interpreted as problematic. There was no statistically significant difference between genders in spite of some differences among groups based on school year or academic achievement.* Conclusions*. We could examine students' perceptions regarding TKM education at a TKM college using DREEM for which validity and reliability were verified. TKM education was perceived relatively poor, but these quantitative indicators suggested which parts of education need improvement. We expect DREEM to be used widely in TKM or traditional medical education field.

## 1. Introduction

Doctors, professionals who are very important resources for a national healthcare system, provide a myriad of medical services to people for the public health and welfare [1, 2]. Professionalism of doctors can be reinforced by individual effort and the educational curriculum of a medical college. Therefore, various organizations affiliated with medical societies have endeavored to improve undergraduate curricula, because the undergraduate program is the most fundamental and important stage in medical education. Since 1983, in which The Korean Society of Medical Education was established, a number of studies on medical education have been conducted and published in Korea [3–8]. Particularly, a survey of students' satisfaction on medical education was conducted in 1995 [9] and a nationwide survey using the Dundee Ready Educational Environment Measure (DREEM) of 40 medical colleges in Korea was conducted in 2013 to identify students' perceptions of the educational environment in these medical schools [8].

Legally, Korea has two distinct and equivalent healthcare systems based on either conventional Western medicine or Korean medicine. To avoid possible confusion in mentioning them, in this paper, we will express system associated with Korean medicine as traditional Korean medicine (TKM), although the official name recognized by the Ministry of Health and Welfare in Korea is just “Korean medicine.”

Regarding TKM, the Institute of Korean Medicine Education and Evaluation (IKMEE) was established in 2004; it has taken charge of evaluating TKM colleges. However, there is no academic society specializing in TKM education but just one department specializing in TKM education at the Pusan National University School of Korean Medicine. Accordingly, the academic and institutional approaches toward TKM education have been very poor compared to those of the conventional Western medical community. Although many studies have reported the importance of the educational environment in medical schools for academic motivation, educational satisfaction, academic achievement, and so on [10–14], students' basic perceptions of TKM education have not been evaluated yet. A few previous studies have addressed certain educational aspects of TKM, such as the study on academic stress responses of TKM college students according to Sasang Constitutions [15] and a survey of students' satisfaction with TKM education [16].

DREEM is a scale for measuring perceptions regarding the education of students majoring in medical-related departments. It was developed in 1997 using a Delphi method for assessing various medical educational institutions throughout the world, based on the Medical Educational Environment Measure (MEEM), Postgraduate Hospital Educational Environment Measure (PHEEM), Surgical Theatre Educational Environment Measure (STEEM), and Anaesthetic Theatre Educational Environment Measure (ATEEM). Also, its validity and reliability were confirmed properly [17, 18]. Since the development of DREEM, it has been widely used with students in medical-related departments such as dentistry, nursing, chiropractic, veterinary, and physiotherapy departments throughout the world [19]. In Korea, Park et al. [8] reported on the nationwide DREEM research of forty undergraduate conventional Western medical colleges and graduate schools. However, there has been no report regarding students of TKM based on the use of DREEM. Therefore, the aim of this study was to investigate and evaluate TKM students' perceptions on TKM education using DREEM and give fundamental information and preliminary data for improving TKM education.

## 2. Materials and Methods

### 2.1. Subjects and Survey Procedure

In Korea, the TKM educational curriculum requires six years to complete. During the first two years, students take general liberal arts courses and attend a few basic Western medical and TKM lectures. Over the next 4 years, they receive professional training in general biomedicine, basic science, conventional medicine, and TKM. Therefore, we conducted this study to target students during this four-year professional training period at a designated TKM college. The survey was conducted over 10 days in June, the latter part of the first semester. One author of this paper, who is also a TKM student, explained protection of anonymity of individual information to all students before the survey. Proper rewards were given to all respondents for their authentic answers.

### 2.2. Dundee Ready Educational Environment Measure (DREEM) Questionnaire

Roff et al. [17] from Dundee University in the UK developed DREEM to evaluate the educational environments and curricula of medical-related departments. It was developed using Delphi method and the expertise among panels of 48 professionals throughout the world, and the validity and reliability of various language versions were statistically verified [18, 21–25]. DREEM consists of 50 items and five subscales: SPL (students' perception of learning, 12 items); SPT (students' perceptions of teachers, 11 items); SAS (students' academic self-perceptions, 8 items); SPA (students' perceptions of atmosphere, 12 items); SSS (students' social self-perceptions, 7 items). Each item has a choice of responses from a bipolar 5-point Likert scale as follows: 4 for* Strongly agree*; 3 for* Agree*; 2 for* Uncertain*; 1 for* Disagree*; 0 for* Strongly disagree*. Nine of the 50 items (items 4, 8, 9, 17, 25, 35, 39, 48, and 50) have negative connotations. Therefore, these items should be coded negatively for statistical analysis.

The developers of DREEM suggested interpretation criteria for the total score, subscale scores, and individual scores for each item [20]. Items with mean scores of 3.5 or greater are viewed as positive, and items with mean scores of 2 or less require closer examination because the score could indicate educational problems. Items with mean scores between 2 and 3 are viewed as somewhat positive but requiring improvement (Table 1). Thus, a higher score suggests a better perception of education. The maximum score using DREEM scale is 200, representing the ideal educational environment [20]. These quantitative and diagnostic aspects of DREEM allow researchers to analyze students' perceptions statistically and identify the strong or weak points of individual educational institutes. Consequently, comparative studies of students' perceptions of certain educational environment may be conducted [26].

In this study, we used the Korean version of DREEM developed in 2015 [8] and modified “medicine” to “TKM” according to the specificity of the target group.

### 2.3. Data Management and Statistical Analysis

Basic descriptive statistics were analyzed, and the *t*-test and one-way ANOVA were used for comparing groups by gender, years in school, and academic achievement. Because this study's aim was to conduct a complete enumeration survey at one college, there was no strong necessity for point or interval estimation, but we conducted hypothesis testing and considered a *p* value lower than 0.05 as statistically significant for the exploratory estimation of TKM college students nationwide. We analyzed data with Microsoft Office Excel 2010 (Microsoft, Redmond, WA, USA) and SPSS 18.0 (SPSS Inc., Chicago, IL, USA).

## 3. Result

### 3.1. Basic Demographic Statistics of Respondents

Totally, 325 (205 males, 118 females, and two respondents who did not report their gender) of all 358 students responded to the survey. Gender, mean grade score data from the previous semester, and the current school years were also acquired for subgroup analysis (Table 2).

### 3.2. The Overall and Subscales Results

The mean of overall scores was 94.65 out of 200. The SPL score was 20.20; SPT, 23.03; SAS, 16.16; SPA, 21.70; and SSS, 13.57. According to the interpretation criteria in Roff's guideline, 9 out of 12 SPL items, 5 out of 11 SPT items, 4 out of 8 SAS items, 7 out of 12 SPA items, and 5 out of 7 SSS items were identified as problem areas. In total, 30 out of 50 items were identified as problematic (Table 3). Questionnaire items and detail scores of each individual item are expressed in Supplementary Material available online at http://dx.doi.org/10.1155/2016/6042967.

### 3.3. Subgroup Analysis

The mean scores for the male group and female group were 95.22 and 93.69, respectively. The mean for male group was higher even though there was no significant difference. Mean scores of SAS, SPA, and SSS were slightly higher for the male group, but there were no statistically significant differences differentiating them from the mean scores for the female group (Table 4).

Comparisons of total and subtotal means are expressed in Table 5 by school year and in Table 6 by academic achievements of the previous semester. A post hoc study showed that significant differences among different school year groups or among different academic achievement groups were not identified distinctly.

## 4. Discussion

### 4.1. Total DREEM Score Analysis and Subscales

We investigated students' perceptions regarding their educational environments with a DREEM survey questionnaire. The total DREEM score of 94.65 out of 200 was quite low compared to studies reported from other countries: European studies usually showed scores from 130 to 153, and some universities in Greece and Germany showed scores from 100 to 110. Asian countries including India, Sri Lanka, Nepal, and Turkey and other mid-eastern countries usually showed scores from 100 to 110, and some other universities showed scores of 130 [19]. The average score from 40 conventional Western medical colleges in Korea was 114 [8]. The TKM college in this study had a similar score to that of medical colleges in Iran [27] and Saudi Arabia [28] and a chiropractic college in Canada [29].

According to the interpretation criteria in Table 2, the SPL subscale can be viewed as negative, and the SPA score suggests that there are many problems to be resolved. Additionally, the SSS score suggests that the educational environment is not good for engaging in study. Moreover, the overall mean score of 94.65 reflects “plenty of problems.” That is, students' perceptions were generally negative, especially on the SPL and SPA subscales; accordingly, urgent improvements are needed.

We examined items with mean scores of 1.5 or less on each subscale. Regarding the SPL subscale, item 13* (the teaching is student-centred)*, item 25* (the teaching overemphasizes factual learning)*, item 44* (the teaching encourages me to be an active learner)*, and item 48* (the teaching is too teacher-centred)* were identified as areas for improvement. Generalizing these findings, we could say that TKM education investigated in this study emphasized passive study like rote learning and was not student-centred. On the SPT subscale, all items scored more than 1.5. On the SAS subscale, item 5* (learning strategies which worked for me before continue to work for me now)* and item 27* (I am able to memorize all I need)* were found to meet the criteria. We found that excessive memorization has been overemphasized. On the SPA subscale, item 12* (this course is well timetabled)*, item 35* (I find the experience disappointing)*, and item 42* (the enjoyment outweighs the stress of the course)* were assessed as meeting the criteria. By these, we learned from these students that they were experiencing stress in college life and dissatisfaction with the curriculum. Finally, on the SSS subscale, responses to item 3* (there is a good support system for students who get stressed)* and item 46* (my accommodation is pleasant)* met the criteria and by these we could find that students cited discomfort in college facilities and felt that support from the college for their stress levels was poor. These results are similar to the study by Chang et al. [15], which reported that the stress levels of students majoring in conventional Western medicine and TKM were higher than those of other college students. In that study, the authors proposed academic characteristics of medical departments causing this result and suggested a correlation between stress of TKM students and academic characteristics of TKM.

### 4.2. Subgroup Analysis by Gender, School Year, and Academic Achievement

In addition, we compared groups according to gender (Table 4), year in school (Table 5), and academic achievement (Table 6). Academic achievement was measured by grades from the previous semester. There was no statistically significant difference between males and females; however, there were significant differences in DREEM scores according to school year and grades. This result is not in agreement with previous studies that cited characteristics of gender, school year, and grades as unimportant to students' satisfaction with their education [9]. In this study, the mean overall score of the male group was a little higher than that of the female group, but it was not significantly different. On the other hand, in a nationwide DREEM study of Western medical students in Korea, the total DREEM score and scores for subscales SAS, SPA, and SSS for the male group were significantly higher than those of the female group [8]. Some previous studies showed no differences between the male and female groups [28], although some cited statistically significant differences based on gender groups [8, 30–34]. Males and females attending the TKM college investigated in this study had similar perceptions on the TKM educational environment, although the study of Kwon et al. [16] showed higher satisfaction levels among female students. A direct comparison of this study and Kwon's is not appropriate because of different measurement scales; however, different results seem to be caused by differences in target populations of the two studies.

For total DREEM scores and subscale scores, freshmen and juniors had higher mean scores than sophomores and seniors resulting from low SPL, SPT, and SPA scores of seniors and low SPT and SPA scores of sophomores. These findings are different from the previous results of Kohli and Dhaliwal [35], which indicated that the DREEM score decreases as the school year increases. Our findings also differ from those of Demirören [36], who reported that the DREEM score is maximized in the third-year student group at the end of the preclinical stage and prior to entering practical sessions. Among Korean studies, the nationwide DREEM survey of Park et al. [8] reported that the total DREEM score and SPL score of sophomores in conventional Western medical colleges were the lowest. Our results also differ from Kwon et al.'s study [16], which indicates that the satisfaction of TKM students decreases as the school year increases. Longitudinal follow-up studies should be conducted to discuss the actual perception changes over time.

In comparing DREEM scores for academic achievement, our result is consistent with that of Park et al. [8] and Mayya and Roff [30]: students with higher academic achievements had more positive perceptions regarding their education. Low-achieving students exhibited more negative perceptions on education in comparison to the total DREEM scores of middle- and high-achieving groups. On the other hand, Abraham et al. [37] reported that low-achieving groups had higher DREEM scores, which they attributed to the educational support system. Previous studies defined academic achievement groups according to three stages (low/middle/high) of self-evaluation [8], or at least one failure experience [30, 37]; in this study, we examined academic achievement based on grades from the previous semester.

### 4.3. Limitations and Further Study

The most important thing in this study interpretation is the following: what we investigated in this study was not the quality of TKM education, but the students' perception in view of education consumers. Of course, students' perceptions are very important in evaluating the quality of education, but in the quality evaluation one should consider other factors such as educational purposes, screening systems, teaching methods, evaluation methods, and curricula.

In this study, we used the Korean version of DREEM [8], but it was not developed by the strict translation methodology and its validity has not been validated yet. To perform further studies using DREEM in Korea, cross-cultural translation/adaptation study should be conducted.

## 5. Conclusion

In this study, we examined students' perceptions regarding TKM education at a TKM college using a DREEM questionnaire for which validity and reliability were verified. Using the quantitative indicators of DREEM, we could perform statistical comparisons and an objective evaluation to diagnose actual educational conditions. In comparison with conventional Western medical education in Korea and other countries, students' perceptions regarding TKM education were relatively poor. To verify causes or trends more clearly, longitudinal studies of a cohort should be considered. We expect DREEM to be used widely in TKM or traditional medical education field throughout the world to evaluate and improve the educational conditions.

## Supplementary Material

This supplementary is the whole results of the survey. Table shows the detail statistics of the students' perception regarding the environment of Traditional Korean Medicine education. According to the Roff's guideline [17], 9 out of 12 SPL items, 5 out of 11 SPT items, 4 out of 8 SAS items, 7 out of 12 SPA items, and 5 out of 7 SSS items were identified as problematic. Means and standard deviations of the individual items and subgroups are expressed.

## Figures and Tables

**Table 1:** An approximate guide to interpret the DREEM scores [20].

*Total and individual scores*	
Total score	
0–50	Very poor
51–100	Significant problem
101–150	More positive than negative
151–200	Excellent
Individual item	
≤2	Problem areas
2-3	Could be enhanced
≥3.5	Real positive points
*Subscales*	
SPL	
0–12	Very poor
12–25	Negatively viewed teaching
25–37	A more positive perception
37–48	Teaching highly regarded
SAS	
0–8	Feelings of total failure
9–16	Many negative aspects
17–24	Feeling more on the positive side
25–32	Confident
SSS	
0–7	Miserable
8–14	Not a nice place
15–21	Not too bad
22–28	Very good socially
SPT	
0–11	Very poor
12–22	Negatively viewed teaching
23–33	A more positive perception
34–44	Teaching highly regarded
SPA	
0–12	Very poor environment
13–24	Many issues need changing
25–36	A more positive attitude
37–48	A good overall feeling

DREEM: Dundee Ready Educational Environment Measure.

SPL: students' perceptions of learning; SPT: students' perceptions of teachers; SAS: students' academic self-perceptions; SPA: students' perceptions of atmosphere; SSS: students' social self-perceptions.

**Table 2:** Basic characteristics of respondents (*N* = 325).

Characteristics	Frequency (%)
Gender	
Male	205 (63.1)
Female	118 (36.3)
No reply	2 (0.6)
Year in school	
First	74 (22.8)
Second	89 (27.4)
Third	80 (24.6)
Fourth	82 (25.2)
Grade score	
4.0≤	31 (9.5)
3.0≤ and <4.0	172 (52.9)
2.0≤ and <3.0	105 (32.3)
No reply	17 (5.2)

**Table 3:** Total results of DREEM survey (*N* = 325).

Subscales of DREEM (number of items)	Max	Min	Mean	SD	Number of problematic items
SPL (12)	44	4	20.20	6.51	9
SPT (11)	38	9	23.03	4.67	5
SAS (8)	32	3	16.16	4.54	4
SPA (12)	38	4	21.70	5.30	7
SSS (7)	23	3	13.57	3.27	5

Total 50 items	175	36	94.65	20.09	30

All responses were acquired using Likert scale (0–4).

DREEM: Dundee Ready Educational Environment Measure.

SD: standard deviation.

SPL: students' perceptions of learning; SPT: students' perceptions of teachers; SAS: students' academic self-perceptions; SPA: students' perceptions of atmosphere; SSS: students' social self-perceptions.

**Table 4:** DREEM score according to gender.

Subscales	Male	Female	*t*	*p* value
SPL	20.19 (7.16)	20.25 (5.26)	−0.10	0.92
SPT	22.79 (4.86)	23.47 (4.36)	−1.25	0.21
SAS	16.42 (4.98)	15.64 (3.63)	1.61	0.11
SPA	22.06 (5.68)	21.07 (4.57)	1.61	0.11
SSS	13.76 (3.44)	13.25 (2.93)	1.40	0.16

Total DREEM score	95.22 (21.98)	93.69 (16.51)	0.71	0.48

DREEM: Dundee Ready Educational Environment Measure.

Statistics are expressed as mean (standard deviation).

**Table 5:** DREEM score according to years in school.

	School years			
	Freshman	Sophomore	Junior	Senior
SPL	21.54 (6.36)^b^	20.65 (6.99)^b^	21.11 (5.93)^b^	17.61 (6.03)^a^
SPT	23.86 (4.15)^bc^	21.46 (4.72)^a^	24.95 (4.41)^c^	22.12 (4.57)^ab^
SAS	16.91 (4.32)^a^	15.82 (4.81)^a^	16.42 (4.14)^a^	15.58 (4.76)^a^
SPA	23.72 (4.88)^b^	20.71 (5.57)^a^	22.22 (4.74)^ab^	20.44 (5.35)^a^
SSS	14.36 (2.95)^a^	13.08 (3.55)^a^	13.32 (3.09)^a^	13.61 (3.30)^a^

Total DREEM score	100.39 (18.87)^c^	91.73 (21.73)^ab^	98.02 (18.61)^bc^	89.36 (19.07)^a^

DREEM: Dundee Ready Educational Environment Measure.

Statistics are expressed as mean (standard deviation).

^abc^Indicators of the homogeneous subsets grouped by Tukey's method.

**Table 6:** DREEM score according to academic achievement.

	Grade scores of the previous semester (academic achievement)		
	Low score	Middle score	High score
	2.0≤ and <3.0	3.0≤ and <4.0	4.0≤
SPL	18.49 (6.78)^a^	20.73 (6.04)^ab^	22.01 (7.25)^b^
SPT	21.93 (5.18)^a^	23.46 (4.14)^a^	23.68 (5.30)^a^
SAS	14.30 (4.39)^a^	17.05 (4.24)^b^	17.35 (5.29)^b^
SPA	20.36 (5.28)^a^	22.28 (5.22)^a^	22.06 (5.40)^a^
SSS	12.53 (3.21)^a^	13.98 (3.15)^b^	14.26 (3.78)^b^

Total DREEM score	87.61 (20.50)^a^	97.50 (18.32)^b^	99.36 (23.43)^b^

DREEM: Dundee Ready Educational Environment Measure.

Statistics are expressed as mean (standard deviation).

^abc^Indicators of the homogeneous subsets grouped by Tukey's method.

## References

[1] Meng K. (2004). Medical education plan for the twenty-first century in korea: hopes and challenges. *Korean Journal of Medical Education*.

[2] Yang E. B., Meng K. H. (2014). Five suggestions for future medical education in Korea. *Korean Journal of Medical Education*.

[3] Choi C. J., Kim J. M., Park Y. G. (2004). Patient-centered attitudes and communication skills in medical students after clerkship. *Korean Journal of Medical Education*.

[4] Ahn D., Park G., Baek K. J. (2007). Academic motivation, academic stress, and perceptions of academic performance in medical students. *Korean Journal of Medical Education*.

[5] Kim S. H., Jeon W. T. (2008). The failure experiences of medical school students: a qualitative study. *Korean Journal of Medical Education*.

[6] Cho A.-R., Han S.-I., Yoon S.-H., Park J. H., Yoo N. J., Kim S. (2010). Methods of effective team-based learning administration and expected effects on medical education. *Korean Journal of Medical Education*.

[7] Kim M. J., Park K. H., Yoo H. H. (2014). Development and validation of the medical student stress scale in Korea. *Korean Journal of Medical Education*.

[8] Park K. H., Park J. H., Kim S. (2015). Students' perception of the educational environment of medical schools in Korea: findings from a nationwide survey. *Korean Journal of Medical Education*.

[9] Kim C. Y., Kim S. M., Seo J. D. (1996). A survey of Students' satisfaction on medical education. *Korean Journal of Medical Education*.

[10] Genn J. M. (2001). AMEE Medical Education Guide No. 23 (Part 1): curriculum, environment, climate, quality and change in medical education—a unifying perspective. *Medical Teacher*.

[11] Genn J. M. (2001). AMEE Medical Education Guide No. 23 (Part 2): curriculum, environment, climate, quality and change in medical education—a unifying perspective. *Medical Teacher*.

[12] Pimparyon P., Roff S., McAleer S., Poonchai B., Pemba S. (2000). Educational environment, student approaches to learning and academic achievement in a Thai nursing school. *Medical Teacher*.

[13] Plucker J. A. (1998). The relationship between school climate conditions and student aspirations. *The Journal of Educational Research*.

[14] Audin K., Davy J., Barkham M. (2003). University quality of life and learning (UNIQoLL): an approach to student well-being, satisfaction and institutional change. *Journal of Further and Higher Education*.

[15] Chang J.-Y., Kim K.-S., Kim B.-S. (2012). Study of on academic stress responses according to sasang constitutions of oriental medicine college students. *Journal of Oriental Neuropsychiatry*.

[16] Kwon S.-W., Shin S.-W., Lim B. (2012). A survey of students satisfaction with education in Traditional Korean Medicine. *Journal of Korean Oriental Medicine*.

[17] Roff S., McAleer S., Harden R. M. (1997). Development and validation of the Dundee Ready Education Environment Measure (DREEM). *Medical Teacher*.

[18] Miles S., Swift L., Leinster S. J. (2012). The Dundee Ready Education Environment Measure (DREEM): a review of its adoption and use. *Medical Teacher*.

[19] Ostapczuk M. S., Hugger A., de Bruin J., Ritz-Timme S., Rotthoff T. (2012). DREEM on, dentists! Students’ perceptions of the educational environment in a German dental school as measured by the Dundee Ready Education Environment Measure: educational environment of a German dental school. *European Journal of Dental Education*.

[20] McAleer S., Roff S. (2001). A practical guide to using the Dundee Ready Education Environment Measure (DREEM). *AMEE Medical Education Guide*.

[21] Dimoliatis I. D. K., Vasilaki E., Anastassopoulos P., Ioannidis J. P., Roff S. (2010). Validation of the Greek translation of the Dundee Ready Education Environment Measure (DREEM). *Education for Health*.

[22] Rotthoff T., Ostapczuk M. S., De Bruin J., Decking U., Schneider M., Ritz-Timme S. (2011). Assessing the learning environment of a faculty: psychometric validation of the German version of the Dundee Ready Education Environment Measure with students and teachers. *Medical Teacher*.

[23] Jakobsson U., Danielsen N., Edgren G. (2011). Psychometric evaluation of the Dundee Ready Educational Environment Measure: Swedish version. *Medical Teacher*.

[24] Tomás I., Casares-De-Cal M. A., Aneiros A. (2014). Psychometric validation of the spanish version of the dundee ready education environment measure applied to dental students. *European Journal of Dental Education*.

[25] Kossioni A. E., Varela R., Ekonomu I., Lyrakos G., Dimoliatis I. D. K. (2012). Students' perceptions of the educational environment in a Greek Dental School, as measured by DREEM. *European Journal of Dental Education*.

[26] Roff S. (2005). The Dundee Ready Educational Environment Measure (DREEM)—a generic instrument for measuring students' perceptions of undergraduate health professions curricula. *Medical Teacher*.

[27] Aghamolaei T., Fazel I. (2010). Medical students' perceptions of the educational environment at an Iranian Medical Sciences University. *BMC Medical Education*.

[28] Al-Ayed I. H., Sheik S. A. (2008). Assessment of the educational environment at the College of Medicine of King Saud University, Riyadh. *Eastern Mediterranean Health Journal*.

[29] Till H. (2004). Identifying the perceived weaknesses of a new curriculum by means of the Dundee Ready Education Environment Measure (DREEM) inventory. *Medical Teacher*.

[30] Mayya S. S., Roff S. (2004). Students' perceptions of educational environment: a comparison of academic achievers and under-achievers at Kasturba Medical College, India. *Education for Health*.

[31] Dunne F., McAleer S., Roff S. (2006). Assessment of the undergraduate medical education environment in a large UK medical school. *Health Education Journal*.

[32] Bouhaimed M., Thalib L., Doi S. A. R. (2009). Perception of the educational environment by medical students undergoing a curricular transition in Kuwait. *Medical Principles and Practice*.

[33] Nahar N., Talukder M. H. K., Khan M. T. H., Mohammad S., Nargis T. (2010). Students' perception of educational environment of medical colleges in Bangladesh. *Bangabandhu Sheikh Mujib Medical University Journal*.

[34] Bakhshialiabad H., Bakhshi M., Hassanshahi G. (2015). Students perceptions of the academic learning environment in seven medical sciences courses based on. *Journal of Advances in Medical Education and Practice*.

[35] Kohli V., Dhaliwal U. (2013). Medical students' perception of the educational environment in a medical college in India: a cross-sectional study using the Dundee Ready Education Environment questionnaire. *Journal of Educational Evaluation for Health Professions*.

[36] Demirören M. (2008). Perceptions of students in different phases of medical education of educational environment: Ankara University Faculty of Medicine. *Medical Education Online*.

[37] Abraham R. R., Ramnarayan K., Pallath V. (2007). Perceptions of academic achievers and under-achievers regrading learning enviroment of Melaka Manipal Medical College (Manipal campus), Manipal, India, using the DREEM Inventory. *South East Asian Journal of Medical Education*.

