# Supplementary material for: Perception Study of Traditional Korean Medical Students on the Medical Education Using the Dundee Ready Educational Environment Measure
Source: Evid Based Complement Alternat Med. 2016 Nov 28;2016:6042967. doi: 10.1155/2016/6042967 (PMC5149675; doi:10.1155/2016/6042967)
Supplement: Supplementary file 1 — This supplementary is the whole results of the survey. Table shows the detail statistics of the students' perception regarding the environment of Traditional Korean Medicine education. According to the Roff's guideline [17], 9 out of 12 SPL items, 5 out of 11 SPT items, 4 out of 8 SAS items, 7 out of 12 SPA items, and 5 out of 7 SSS items were identified as problematic. Means and standard deviations of the individual items and subgroups are expressed. [file 6042967.f1.docx]

Supplementary. DREEM items and individual results.

| Items (possible max total score: 200) | Mean | SD | Missing |
| --- | --- | --- | --- |
| SPL (12 items, possible max score: 48) | | | |
| 1. I am encouraged to participate in class | 2.25 | 0.95 | 2 |
| 7. The teaching is often stimulating | 1.59 | 0.86 | 1 |
| 13. The teaching is student centred | 1.33 | 0.84 | 1 |
| 16. The teaching is sufficiently concerned to develop my competence | 2.22 | 0.82 | 0 |
| 20. The teaching is well focused | 2.04 | 0.82 | 1 |
| 22. The teaching is sufficiently concerned to develop my confidence | 1.63 | 0.87 | 0 |
| 24. The teaching time is put to good use | 1.68 | 0.81 | 0 |
| *25. The teaching over-emphasizes factual learning | 1.07 | 0.90 | 0 |
| 38. I am clear about the learning objectives of the course | 1.87 | 0.81 | 0 |
| 44. The teaching encourages me to be an active learner | 1.47 | 0.90 | 0 |
| 47. Long-term learning is emphasized over short term | 1.54 | 1.10 | 1 |
| *48. The teaching is too teacher-centred | 1.50 | 0.83 | 0 |
| SPL subtotal (max/min = 44/4) | 20.20 | 6.51 |  |
| SPT (11 items, possible max score: 44) | | | |
| 2. The teachers are knowledgeable | 2.78 | 0.71 | 0 |
| 6. The teachers are patient with patients | 2.32 | 0.70 | 2 |
| *8. The teachers ridicule the students | 2.25 | 0.85 | 0 |
| *9. The teachers are authoritarian | 1.76 | 0.90 | 1 |
| 18. The teachers have good communications skills with patients | 2.24 | 0.63 | 10 |
| 29. The teachers are good at providing feedback to students | 1.88 | 0.84 | 0 |
| 32. The teachers provide constructive criticism here | 2.01 | 0.81 | 0 |
| 37. The teachers give clear examples | 1.87 | 0.84 | 0 |
| *39. The teachers get angry in class | 1.86 | 0.98 | 0 |
| 40. The teachers are well prepared for their classes | 2.28 | 0.80 | 0 |
| *50. The students irritate the teachers | 1.80 | 0.94 | 1 |
| SPT subtotal (max/min = 38/9) | 23.03 | 4.67 |  |
| SAS (8 items, possible max score: 32) | | | |
| 5. Learning strategies which worked for me before continue to work for me now | 1.48 | 1.08 | 0 |
| 10. I am confident about passing this year | 3.09 | 0.91 | 0 |
| 21. I feel I am being well prepared for my profession | 1.68 | 0.90 | 2 |
| 26. Last year's work has been a good preparation for this year's work | 2.35 | 0.86 | 0 |
| 27. I am able to memorize all I need | 1.22 | 0.86 | 0 |
| 31. I have learned a lot about empathy in my profession | 2.02 | 0.83 | 1 |
| 41. My problem-solving skills are being well developed here | 2.00 | 0.89 | 0 |
| 45. Much of what I have to learn seems relevant to a career in medicine | 2.31 | 0.90 | 1 |
| SAS subtotal (max/min = 32/3) | 16.16 | 4.54 |  |
| SPA (12 items, possible max score: 48) | | | |
| 11. The atmosphere is relaxed during the ward teaching | 1.92 | 0.65 | 17 |
| 12. This school is well timetabled | 1.43 | 0.87 | 2 |
| *17. Cheating is a problem in this school | 2.01 | 1.10 | 0 |
| 23. The atmosphere is relaxed during lectures | 2.14 | 0.77 | 0 |
| 30. There are opportunities for me to develop interpersonal skills | 2.08 | 1.06 | 1 |
| 33. I feel comfortable in class socially | 2.23 | 0.97 | 1 |
| 34. The atmosphere is relaxed during seminars/tutorials | 2.38 | 0.84 | 1 |
| *35. I find the experience disappointing | 1.07 | 0.81 | 0 |
| 36. I am able to concentrate well | 1.79 | 0.83 | 0 |
| 42. The enjoyment outweighs the stress of studying medicine | 1.46 | 0.97 | 1 |
| 43. The atmosphere motivates me as a learner | 1.60 | 0.84 | 1 |
| 49. I feel able to ask the questions I want | 1.61 | 0.98 | 0 |
| SPA subtotal (max/min = 38/4) | 21.70 | 5.30 |  |
| SSS (7 items, possible max score: 28) | | | |
| 3. There is a good support system for students who get stressed | 0.90 | 0.80 | 0 |
| *4. I am too tired to enjoy this course | 1.79 | 1.05 | 0 |
| 14. I am rarely bored on this course | 1.72 | 1.08 | 0 |
| 15. I have good friends in this school | 3.06 | 0.77 | 0 |
| 19. My social life is good | 2.77 | 0.72 | 0 |
| 28. I seldom feel lonely | 1.88 | 1.02 | 0 |
| 46. My accommodation is pleasant | 1.45 | 0.99 | 2 |
| SSS subtotal (max/min = 23/3) | 13.57 | 3.27 |  |
| DREEM total (max/min = 175/36) | 94.65 | 20.09 |  |
| DREEM: Dundee Ready Educational Environment Measure  SD: standard deviation  SPL: Students’ perceptions of learning; SPT: Students’ perceptions of teachers; SAS: Students’ academic self-perceptions; SPA: Students’ perceptions of atmosphere; SSS: Students’ social self-perceptions  *Negative statements. They should be coded negatively to perform statistical analysis. | | | |
